# Insightful Practice: a robust measure of medical students’ professional response to feedback on their performance

**DOI:** 10.1186/s12909-015-0406-2

**Published:** 2015-08-01

**Authors:** Douglas Murphy, Patricia Aitchison, Virginia Hernandez Santiago, Peter Davey, Gary Mires, Dilip Nathwani

**Affiliations:** 1 Quality, Safety and Informatics Research Group, Population Health Sciences, University of Dundee, Mackenzie Building, Kirsty Semple Way, Dundee, DD2 4BF UK; 2 Nursing, Midwifery & Allied Health Professionals Research Unit (NMAHP), Innovation Park, University of Stirling, Stirling, FK9 4NF UK; 3 Ninewells Hospital, Dundee, DD1 9SY UK

**Keywords:** Feedback, Continuous professional development, Patient safety, Professionalism, Remediation

## Abstract

**Background:**

Healthcare professionals need to show accountability, responsibility and appropriate response to audit feedback. Assessment of *Insightful Practice* (engagement, insight and appropriate action for improvement) has been shown to offer a robust system, in general practice, to identify concerns in doctors’ response to independent feedback. This study researched the system’s utility in medical undergraduates.

**Methods:**

*Setting and participants*: 28 fourth year medical students reflected on their performance feedback. Reflection was supported by a staff coach. Students’ portfolios were divided into two groups (n = 14). Group 1 students were assessed by three staff assessors (calibrated using group training) and Group 2 students’ portfolios were assessed by three staff assessors (un-calibrated by one-to-one training). Assessments were by blinded web-based exercise and assessors were senior Medical School staff.

*Design*: Case series with mixed qualitative and quantitative methods. A feedback dataset was specified as (1) student-specific End-of-Block Clinical Feedback, (2) other available Medical School assessment data and, (3) an assessment of students’ identification of prescribing errors.

*Analysis and statistical tests*: Generalisability G-theory and associated Decision D- studies were used to assess the reliability of the system and a subsequent recommendation on students’ suitability to progress training. One-to-one interviews explored participants’ experiences.

*Main outcome measures*: The primary outcome measure was inter-rater reliability of assessment of students’ *Insightful Practice*. Secondary outcome measures were the reaction of participants and their self-reported behavioural change.

**Results:**

The method offered a feasible and highly reliable global assessment for calibrated assessors, G (inter-rater reliability) > 0.8 (two assessors), but not un-calibrated assessors G < 0.31. Calibrated assessment proved an acceptable basis to enhance feedback and identify concern in professionalism. Students reported increased awareness in teamwork and in the importance of heeding advice. Coaches reported improvement in their feedback skills and commitment to improving the quality of student feedback.

**Conclusions:**

*Insightful practice* offers a reliable and feasible method to evaluate medical undergraduates’ professional response to their training feedback. The piloted system offers a method to assist the early identification of students at risk and monitor, where required, the remediation of students to get their level(s) of professional response to feedback back ‘on track’.

**Electronic supplementary material:**

The online version of this article (doi:10.1186/s12909-015-0406-2) contains supplementary material, which is available to authorized users.

## Background

The public expect their healthcare staff to be professionals. It is becoming clear that the drive for Quality Improvement in healthcare needs to extend its current scope beyond a focus on systems and processes to include the training and professionalism of those who care for them [1–3]. The public both deserve and demand that those who provide care for patients are accountable and ensure that the public is protected [3]. To achieve this, healthcare teams need their multi-disciplinary membership to develop and maintain their individual standards of professionalism from the outset of their careers. Any problems need to be highlighted as early as possible to allow intervention, support and remediation.

This is not simple – there are problems.

First, measurement of professionals may be interpreted as performance management, aimed to achieve organisational goals, or to identify ‘bad apples,’ rather than supporting individuals and teams to enhance and maintain their expertise [1]. Second, context specificity of a multitude of professional work-roles and circumstances would appear to require a multitude of different validated tools, thus making implementation problematic and potentially unworkable [4]. Third, workplace measurement may stand accused of ‘hoop jumping’ and be of limited or no value [5]. Fourth, the fitness of face-to-face appraisal to extend its expected role from that of supporting professional development to identify ‘bad apples’ has been questioned [6]. Lastly, there is a need to ensure that when professionals give undertakings to improve their practice these provide adequate protection to patients and that the practitioners involved have shown insight and have recognised the steps needed to limit their practice or provide remediation [7].

Innovation is needed. An early warning system for professionals to highlight when they are in danger of, or have wandered ‘off track’ in their level(s) of professionalism is desirable and would offer early opportunity for remediation. *Insightful Practice* has been defined as professionals demonstrating professional responsibility and accountability by demonstrating their appropriate levels of engagement, insight and action when presented with credible and independent feedback on individual and/or team performance [6]. Originally developed to support professional revalidation and appraisal, the system offers a robust method to allow professionals to use a suite of contextually driven independent feedback to supplement self-assessment and support plans for quality improvement. Importantly, the plans for improvement benefit from peer discussion to help promote insight and set objectives for improvement [6].

This study set out to explore the utility of the system of *Insightful Practice* to measure professional response to feedback in a group starting their professional careers – medical students. Study processes and participants’ reflections and actions were captured using a web-based portfolio – Tayside Insightful Practice Portfolio (TIPP) [8]. If successful, the system would help offer the earliest identification of those in need of remediation and provide a platform for monitoring and sustaining appropriate professional response and commitment to career long continuous improvement.

The study aimed to answer the following principal research questions:

1. Can the measure of Insightful Practice discriminate (measure reliably) between students in their appropriate professional response to feedback on their performance?
2. Can the measure of Insightful Practice offer a reliable recommendation on students’ progression in training?
3. How supportive were students and staff coaches of the study’s system in its capacity to support students’ professional development and allow quality assurance of the medical school’s support and provision of feedback to students?

## Methods

This was based on a case series design to track medical students’ performance outcomes over time in response to a suite of specified feedback. Recruited medical students’ suite of data included:

1. End-of-Block feedback reports provided to students following each rotational clinical attachment: (Additional file 1)
2. e-GRID exercises: a web-based grid recognition system developed for the study which, in this application, focused on prescribing errors within hospital in-patient prescribing charts – Spot-the-Error (see Additional file 2).
3. Other available Medical School assessment data: online exams, OSCE, PowerPoint presentations, case presentations, viva assessments, mini-CEX, case based discussions, pre and post course assessment results and any record of other assessments completed during four week blocks (clinical attachments) in 4th Year.

Participants’ reflection on their feedback was assisted by a provided generic Feedback Improvement Tool (FIT) (see Additional file 3). Following students’ self-reflection they received a coaching interview from a member of senior Medical School staff to help support students’ *Insightful Practice* by helping them demonstrate appropriate reaction to their collected feedback. Students’ success in showing *Insightful Practice* was subsequently assessed by three anonymous and blinded Medical School assessors (see Additional file 4). The reliability of this assessment of *Insightful Practice* and subsequent recommendation on students’ suitability for progress of training was investigated using Generalisability G-theory [9–11]. Decision (D) studies were conducted to determine the number of assessors required to achieve an inter-rater reliability of 0.8, as required for a high-stakes assessment [9, 10].

Following participation in the above, coaches and students were interviewed to explore their experiences of participating in a new and unfamiliar system to steer future student development and professionalism.

### Quantitative methods

#### Participants and sample size calculation

Forty-three volunteer fourth year medical students within University of Dundee in Scotland were recruited for entry into the study. Twenty-eight students (65 %) completed the study. Two information meetings were held, after which medical students signed a register to confirm interest in participating. A consent form was sent to each with a covering letter and study information sheet. The power calculation was based on Fisher’s ZR transformation of the intra-class correlation co-efficient [9]. Given a required reliability Intraclass Correlation Coefficient (ICC) R of 0.8 for a high-stakes assessment of portfolios, specified standard error of the reliability of 0.1 and three assessors of each subject, Fisher’s ZR transformation specified a minimum of 15 subjects [9].

#### Performance measures and data collection

Study processes were facilitated by a website called Tayside Insightful Practice Portfolio (TIPP) developed to administer, collect and assess all participant data, [8] making the allocation of tasks and feedback feasible. Students were asked to reflect on their suite of collected data (Table 1) prior to a coaching session with an appointed University staff coach.

**Table 1:** **Summary of tools used and process followed** ^a^

Method of feedback	Application	Source	Prepared by
Written	End-of-Block Feedback (see Additional file 1)	Existing Medical School Feedback	Medical School
‘Spot-the-Error’ prescribing application of e-GRID Web-based interactive exercises	Examples of Patient Prescribing Charts^a^ (see Additional file 2)	Developed for Study	Study Researchers
EMI	Extended matching item format test designed to assess the clinical application of knowledge base	Existing Medical School Feedback	Medical School
OSCE	12 station clinical OSCE including consultations, examinations, procedures and data interpretation.	Existing Medical School Feedback	Medical School
Case Presentation	During Block	Existing Medical School Feedback	Medical School
PowerPoint presentation		Existing Medical School Feedback	Medical School
Mini-CEX	A structured observation and feedback form used to guide evaluation of student’s consultation and/or examination skills	Existing Medical School Feedback	Medical School
Case Based Discussion	A structured feedback form used to guide assessment of discussion regarding a patient seen in general practice	Existing Medical School Feedback	Medical School
Viva Assessment	Oral examination	Existing Medical School Feedback	Medical School

The reliabilities of individual tools are not reported here

^a^Data on 32 drug charts were made available by pharmacists at NHS Tayside. These consisted of two drug prescription charts for each attached medical student block. Other tools used are available to medical students to include when considering their suite of individual feedback

#### Study steps: reflection, Appraisal and assessment

**Step 1**: (September 2011 – June 2012)

*Collection of specified feedback*:

Study participants were provided with data via the study website including:

- End-of-Block (Clinical Specialty) Feedback (see Additional file 5)
- ‘Spot-the-Error’ e-GRID web-based recognition application on prescribing errors (see Additional file 2)
- Other individually available Medical School feedback including: online exams, OSCE, PowerPoint presentations, case presentations, viva assessments, mini-CEX, case based discussions, pre and post course assessment results and block assessment results.

**Step 2**: (September 2011 – June 2012)

*Student reflection on feedback and setting personal objectives for improvement prior to availability for face-to-face coaching interview*: (see Additional file 3)

Having reflected on his or her performance feedback, participants used a generic reflective template used in the earlier GP study now named – Feedback Improvement Tool (FIT) [6]. FIT is designed to support personal reflection by docking with any suite of independent contextualized feedback data. For the study, FIT consisted of four 7-point Likert scales to rate each source of feedback data as having:

1. Highlighted important issues
2. Demonstrated concern in performance
3. Led to planned change
4. Given valuable feedback

Students then wrote a free-text commentary and framed any planned actions as **S**pecific, **M**easurable, **A**chievable, **R**elevant and **T**imed (SMART) objectives [12].

**Step 3**: (February 2012 – June 2012)

Students then received a face-to-face coaching interview from one of the eleven study staff coaches recruited from senior Medical School staff (Table 2). These coaching interviews offered students the opportunity for facilitated reflection and possible amendment of their prepared personal objectives. Following these face-to-face coaching sessions students finalised their personal objectives for improvement (see Additional file 3).

**Table 2:** **Rating questions completed by students and coaches**

Question	Rating scale	Completed by
**2a. Feedback Improvement Tool FIT**. [6]	Likert 1-7*^1,2^	
Source of feedback highlighted:		▪ Student participant
1. Important issues		▪ Face-to-face coach (post-coaching session)
2. Concern in my performance		
3. Led to planned change		
4. Gave valuable feedback		
**2b. Assessment of Insightful Practice Template (AIP)** [6]	Likert 1-7*^1^	▪ Anonymous coach assessor (post-coaching session)
Student demonstrated:		
1. Satisfactory engagement with the TIPP process		
2. Insight into feedback provided on performance		
3. Plans for appropriate action where applicable		
4. Engagement, insight and action (global rating of *insightful practice*)		
5. Suitability for student progression recommendation	Binary yes/no	▪ Anonymous coach assessor (post-coaching session)

(2a) completed by student participants (pre-coaching session) and rating questions

(2a, 2b) completed by anonymous web-based portfolio assessors (post coaching session)

*1Likert scale descriptors (1–7): (1) strongly disagree; (3) disagree; (5) agree; (7) strongly agree

*2The AIP assessment has now been included in FIT as a self-assessment (see in Additional file 3)

**Step 4**: (June 2012 – July 2012)

An anonymous post-coaching session assessment of participants’ level of *Insightful Practice* was completed post coaching session by two groups of three additional anonymous coaches (see Additional file 4). Each assessor group marked 14 students’ portfolios.

*Group 1* assessors attended a group training session that included a calibration exercise. *Group 2* assessors received an individual one –to –one training session, but were not calibrated as a group due to other commitments.

These coaches rated the students using an Assessment of *Insightful Practice* template (AIP) with four 7-point Likert scales (see Additional file 4). These related to students’ *engagement* with the process*, insight* into data collected, *planning of appropriate action* in response, and a global rating of their engagement, insight and action as a marker of students’ *Insightful Practice*. Additionally, the coach was asked to assess whether the student should be recommended as progressing satisfactorily without further opinion (Table 2).

**Step 5**: June 2012 – August 2012

One-to-one interviews were conducted with 5 students and 9 coaches.

#### Reliability

The reliability of the *Insightful Practice* measurement tool was calculated using Generalisability G-theory by a web-based anonymous marking exercise post coaching interview [9–11]. Anonymous assessors were recruited from Medical School senior staff (n = 6). Two groups of assessors (n = 3) each marked 14 student portfolios (raters nested within group). Reliabilities (internal consistency and inter-rater) of anonymous assessor decisions for Assessment of *Insightful Practice* (AIP) Questions 1–3, and inter-rater reliabilities for AIP Questions 4 and 5 were assessed by Generalisability G-theory using GENOVA and G String IV software [10, 11].

For the analysis of Internal Consistency, Student (S) was treated as the facet of differentiation, Question (Q) as the facet of generalization and Rater (R) (assessor) as a fixed facet. Internal consistency does not account for error contributed by different assessors and can inflate reliability results where raters are used within an assessment [9]. As a result, inter-rater reliability was calculated. For the analysis of inter-rater reliability, Student (S) was the facet of differentiation, Rater (R) (assessor) the facet of generalisation and Question (Q) was treated as a fixed facet. Decision (D) studies were conducted to determine the number of assessors required to achieve an inter-rater reliability of 0.8, as required in high-stakes assessment [9] 95 % confidence intervals for reliabilities (Questions 4 and 5) were calculated using Fisher’s ZR transformation [9].

### Qualitative methods

#### Data collection and participants

Data collection was carried out during June-August 2012. Semi-structured interviews were conducted either face-to-face or by telephone, with the support of a topic guide covering key aspects of interest to the study. Informed consent was obtained from all participants beforehand. Interviews were digitally recorded and transcribed verbatim for analysis.

Five students were locally available and agreed to be interviewed, representing approximately 18 % (5/28) of all students who completed participation in the pilot. Difficulties recruiting students were experienced because around the time of the study students were preparing for exams and then, subsequently, leaving for summer electives.

Nine coaches were interviewed, representing 82 % of the total number of coaches (9/11).

#### Data analysis

Data were analysed using the ‘Framework’ approach [13]. Data were systematically organised and summarised under identified themes, with due attention given to the topics that guided the interviews with participants. Themes were categorised by a qualitative researcher who reported to the other authors on this aspect of the analysis. Analysis charts assisted in identifying patterns and relationships within the data leading to the emergence of the research findings.

### Ethical approval

Formal application and submission of the research proposal was made and ethical approval granted for all of the work contained in this paper by University of Dundee Research Ethics Committee. Participants gave informed consent before participating.

## Results

Forty-three student volunteers were recruited to the study. 28 (65 %) completed the study.

### Quantitative results

#### Descriptive statistics

There was no significant difference between Group 1 and 2 assessors’ overall grand mean of scores that they had awarded to each of their respective groups of students for AIP question 4 (assessors’ global rating of students’ Insightful Practice) – with identical values of 4.43.

Un-calibrated assessors showed a wider range than calibrated assessors in their global ratings of students’ Insightful Practice (AIP Q4):

- Group 1 (calibrated) assessors’ range was 4.07 – 4.64 (1–7 scale).
- Group 2 (un-calibrated) assessors’ range was 3.64 – 5.36 (1–7 scale).

Overall range of scores for Group 1 were (1–7) and for Group 2, (2–7).

2/14 (14.3 %) of students were considered by Group 1 assessors as unable to recommend for progression without further consideration (AIP question 5; Table 2). 1/14 (7.14 %) of students were unable to be recommended by Group 2 assessors (AIP question 5; Table 2)

#### Reliability of insightful practice

Internal consistency: The Assessment of *Insightful Practice* marking tool demonstrated high internal consistency (Cronbach’s alpha) for both groups. Based on the marking by the three calibrated assessors of AIP questions 1–3 gave a Cronbach alpha of 0.96. Internal consistency based on the marking by the study’s three un-calibrated assessors marking of AIP questions 1–3 gave a similarly high score for Cronbach alpha of 0.92.

High values for Cronbach alpha confirms the AIP assessment questions of engagement, insight and action all correlate highly with each other and so reassures on the consistency of the questions within the overall measure of *Insightful Practice*.

Inter-rater Reliability: High inter-rater reliability was shown by the calibrated group of assessors (Group 1) across questions (1–3) engagement, insight and action, question 4 (global rating) and a dichotomous question (5) on suitability for training progression (Table 3a). There was low inter-rater reliability shown by the un-calibrated (Group 2) assessors across all questions (Table 3b).

**Table 3:** **Inter-rater Reliability of Assessment of Insightful Practice (AIP) Questions**

3a - GROUP 1 (Calibrated Assessors)			
	AIP questions 1–3 (engagement, insight and action) 1-7 scale	AIP question 4 (global assessment) 1-7 scale	AIP question 5 (Dichotomous assessment on suitability for progression recommendation)
Number of Raters	Inter-Rater Reliability (G)^b^	Inter-Rater Reliability (ICC)^a^ (G)^b^ (95 % confidence interval)^c^	Inter-Rater Reliability (ICC)^a^ (G)^b^ (95 % confidence interval)^c^
**1**	**0.76**	**0.73** (−)	**0.75** (−)
**2**	**0.87**	**0.84** (0.57-0.95)	**0.85** (0.59-0.95)
**3**	**0.91**	**0.89** (0.73-0.96)	**0.9** (0.76-0.96)
**3b-** GROUP 1 (Assessors not calibrated)			
**GROUP 2 (Assessors NOT Calibrated)**	**AIP questions 1–3 (engagement, insight and action) 1-7 scale**	**AIP question 4 (global assessment) 1-7 scale**	**AIP question 5 (Dichotomous assessment on suitability for progression recommendation)**
(n) Raters	Inter-Rater Reliability (G)^b^	Inter-Rater Reliability (ICC)^a^ (G)^b^	Inter-Rater Reliability (ICC)^a^ (G)^b^
**1**	0.33	0.18	0.16
**2**	0.5	0.31	0.28
**3**	0.6	0.4	0.37

^a^Intraclass Correlation Coefficients (ICCs) are G-coefficients when you have a one facet design (rater)

^b^Inter-rater reliability is the extent to which one rater’s assessments (or when based on multiple raters, the average of raters’ assessments) are predictive of another rater’s assessments

^c^95 % confidence intervals for reliabilities (ICCs) were calculated using Fisher’s ZR transformation which is dependent on raters (5) with a denominator value of (n-1), so cannot be calculated when only one rater. (Streiner and Norman, [9])

Analysis of variance (ANOVA) tables; components of variance; and formulae used for all calculations including statistical facets of differentiation, generalization and fixed facets are given in Additional file 5.

### Qualitative results

Students’ coaching sessions took place February 2012 to June 2012. Coaches were largely in agreement that TIPP (the study system) was/or had the potential to be useful in helping students to engage in the process of reviewing and in helping them reflect on all of their feedback. This included feedback received at their coaching session. Both of which helped facilitate *Insightful Practice*. Cautiousness was expressed, however, about the extent to which complete confidence could be placed in the impact of TIPP at this stage, given, for example:

- technical and design issues associated with the website
- concerns about the quality of End-of-Block feedback
- limitations possibly inherent in having only a single coaching session
- having participant students who were, possibly, more motivated than others.

Overall, coaches considered the TIPP pilot project feasible to implement in terms of time allocation and resources. Overall, students considered TIPP feasible, noting the easiness of the on-line system and limited time required for associated tasks.

As a means of evaluating qualitative study findings, we considered data under Barr’s adaptation of Kirkpatrick’s evaluation model levels 1–4 [14].

*Level 1—Learner’s reactions*

Students were predominantly positive about their coaching sessions and generally valued the experience of meeting with someone within a positive context mid-way through the year to discuss their feedback and to reflect on the performance and progress.

*‘So I feel it’s nice to have something sort of mid-way through the year just to know you’re roughly on track or you’re going a wee bit off.’ [Student 2, Male]*

One student added some cautionary notes regarding the acceptability and feasibility of TIPP, particularly highlighting that, for students, 4th year was already a demanding year, work-wise. Given this, he reflected that students may view the obligation to participate in additional self-reflection as something being done just for the sake of it, unless they were convinced that this process was something of value to them. In turn, his view was that this depended largely on students’ perceptions of how meaningful their feedback was.

*‘I think it is feasible. I would be wary of adding more enforced reflection on to students because there is a tendency to see that as a hoop-jumping exercise and not using TIPP for the value that it could hold, so I think that there does need to be some sensitivity in adding further paperwork particularly in fourth year which is a very work-heavy year. I don’t think it’s unfeasible as long as there’s an acceptable value to it and I think that, in large part, this relies on getting meaningful feedback from the blocks in order to be able to reflect on it.’* [Student 3, male]

*Level 2a—Modification of attitudes and perceptions*

*Level 2b—Acquisition of knowledge and skills*

Coaches reported that most students had completed their reflective templates prior to their coaching session, but while some had fully completed these competently, others’ reflections appeared vague and lacking in detail.

Some coaches expressed the view that the collation of feedback within TIPP had, for them, brought sharply into focus their concerns about the quality of feedback given to students during their fourth year. They were critical about the lack of depth and limited range of feedback sometimes given to students.

*‘I like it [feedback] coming together, but I thought what I got, in terms of what I read from the various blocks, a bit disappointing. I don’t think our tutors are providing the depth, breadth of feedback that I would want for my block. I think it is very superficial and scratchy.’ [Coach 1]*

In considering student feedback, coaches’ attention was drawn to the possible differences between tutors’ and students’ expectations of feedback. Where students’ personal ambitions focused on continued development and high achievement they may have expectations that feedback would contribute effectively to this process; in contrast, clinicians and tutors may expect to spend limited time and resources preparing End-of-Block feedback, unaware of the importance that students attached to it.

*‘I think probably most people feel that you’re just saying that the students have turned up and therefore that’s ok, and you’re really only expected to comment if there’s a major concern, if you think the student had a measurably weaker block, [but] that may not be what the students are looking for.’ [Coach 8]*

Students, reported that participation provided better feedback than they had received previously, which was described as ‘vague’, ‘limited’ and ‘inadequate’,

*‘I thought that [reflective template:* FIT*] was useful because it made you focus more on what you had done the previous block and gave you a bit more in-depth feedback, because all you’d normally get from the supervisor would be sort of two lines, either’very good’ or ‘needs to improve’.’ [Student 2, Male]*

*‘I suppose it [reflective template:* FIT] *was a useful process in focusing us and helping structure our reflections on the feedback. [Student 3, Male]*

Coaches perceived that, given the lack of direct and formal feedback, students tended to draw on verbal feedback and personal reflections about their performance as a basis for identifying personal learning objectives, but this meant that learning objectives were often vague and focused more on general areas of personal development; for example, time management and communication skills. Some coaches described how coaching sessions could, in these instances, provide a useful focus for identifying and prioritising specific aspects of students’ learning and development within a SMART framework [12]. Paradoxically, perhaps, lack of evidence of effective reflection by students could open up more opportunities for dialogue around their feedback, performance and learning objectives.

*‘They [students] tend, with the [learning] plan, tended to have left it quite generalised and it was helping them finish it off properly in terms of, so, what are you going to do specifically, within this specific time period, that’s going to be helpful for you, rather than “I will read more around something at some point”?’ [Coach 3]*

*‘I think one of the issues [students] had was lack of quality feedback (…) and they would talk about the verbal feedback they’d had and experiences they’d had on the block, so we’d actually pull that out and be able to use some of the information to help them inform their learning objectives for the future.’ [Coach 5]*

*‘I remember one student who had very little [reflection] (…) so it was quite good that you had something to build on and something to tweak. In fact the ones who hadn’t done it quite so well were a bit more fruitful than the ones who had just done it in the manner I thought was outstanding really.’ [Coach 6]*

Students generally found the support and feedback from their coaching interview helpful.

*‘The thing about it [TIPP] is you can’t really use feedback from your tutors but you can from your coach.’ [Student 1, Female]*

*‘(…) any worries I had she [coach] could discuss with me and I felt quite relieved actually after visiting her that, you know, I was maybe a bit anxious about what lay ahead but she kind of calmed me down, it was like “You’re doing fine, just keep going”. So I took quite a lot of reassurance from meeting her and I found it a benefit perhaps as part of the [TIPP] project but, personally, I found it really useful to meet her.’ [Student 4, Female]*

*Level 3—Change in behaviour*

From a wider perspective, one student reflected that the educational value of reflecting on and responding appropriately to feedback extended to her long-term professional development.

*So, I guess any feedback that I get this year and got last year is feeding into my career and I’d be a fool I think to not heed somebody’s feedback or somebody’s comment because it could be something really important (…) And you’re part of a whole team, I see myself, I’m not just a student, I’m part of the team as well, so I think heeding people’s advice and heeding people’s feedback is a mature way to act.’ [Student 4, Female]*

Some coaches reflected on the contribution that participation in TIPP had made to their own learning and development, and to gaining insight into their own practice, particularly in relation to giving feedback and being sensitive to students’ learning styles and experiences.

*I found it time-consuming but I enjoyed it because I learned about things I’m not familiar with.’ [Coach 1]*

*‘I mean, it’s the students’ call as to what the benefits [of TIPP] would be but I think some of the benefits are around improving our ability as tutors and trainers and teachers to give feedback and, you know, with a lot of the students (…) I would ask them about what was good feedback and what was bad feedback and hearing what they had to say was chilling in terms of some of the feedback that I give and how I would want to improve.’ [Coach 6]*

*‘I guess in some respects it was a useful process for myself in terms of considering learning styles and other postgraduate trainees rather than just undergraduates. I thought it was interesting for me to consider how my feedback may be then used in other ways, considering whether my feedback is adequate or if I should be improving it.’ [Coach 4]*

*Level 4a—Change in organisational practice*

The Medical School is considering change in organisational practice, with alignment of TIPP to supporting students with professionalism concerns and also with testing the TIPP Spot-the-Error resources as core to support prescribing skills in 4th Year and reducing prescribing errors made by our graduates. This could provide an opportunity for evaluation at Level 4 in the future.

*Level 4b—Benefits to clients or patients*

This study was not designed to evaluate outcomes at level four.

## Discussion

### Summary

This study demonstrates that the measure of *Insightful Practice,* by calibrated assessors, offers a reliable measure of medical students’ professionalism and allows a robust recommendation on the suitability of progression in their training. This assessment, at the outset of their careers, is based on the assessment of evidence based on students’ responsibility and accountability when presented with credible and independent evidence on their performance. Participant students and staff coaches were supportive of the system in its capacity to support students’ professional development and to offer a method to quality assure the quality of the medical school’s provided feedback to students.

### Context

Methods used in this study have previously shown the measurement of *Insightful Practice* to offer a robust basis for decisions to underpin professional revalidation in general practice. Success in the undergraduate sphere, in this study, suggests *Insightful Practice* may offer a generic basis for educational support to benefit professional expertise and quality improvement and, where needed, offer a supportive system of remediation and a judgement on final outcomes for patient safety.

### Interpretation

**Fig. 1:**
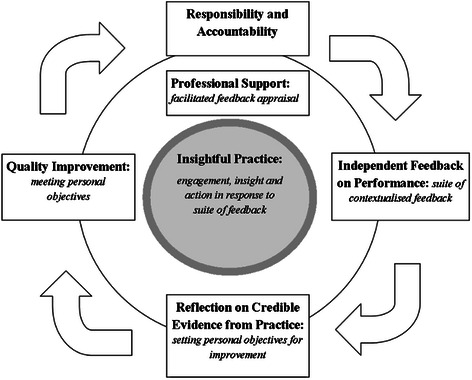
Cycle of insightful practice

The proposed role of *insightful practice* to act as the hub within a continuous cycle to generate, monitor and maintain objective evidence of personal responsibility and accountability for quality improvement has already been demonstrated (Fig 1).

Undertaking a professional career in healthcare is difficult and fraught with challenges including work, personal and health matters. Rather than searching for a system to find the bad apple, we should consider how an early warning system to support professionalism and expertise, during both the training and practice stages of career, could be developed to identify and target resources on those at risk of becoming ‘bad apples’ and so prevent risk to patients. A degree of remediation is probably needed by all throughout career. It is the size, seriousness and urgency of help needed that is the key point. Remediation limited to the identification and support of those already demonstrating incompetent skills, or dangerous attitudes may be attractive by limiting the numbers generated. This approach, however, clearly fails patients and professionals’ part way on the road to disaster and also misses the opportunity to support general quality improvement in expertise. There is a need to accept that we start by measuring professionals doing a privileged and unique role. We assert that the recognition of reduced standards of professionalism is more easily recognised than the many facets and attributes of professionals. The practice of healthcare is a privilege. Given that at the start of career on entry into training one is ‘on track’, there is a strong argument that future monitoring of satisfactory progress throughout both training phase and career is desirable and offers practitioners confirmation of their ongoing professionalism, self-worth and standing with their patients. The measure of *Insightful Practice* offers individuals and teams a system to cross-check their self-assessment of plans for improvement as well as allowing independent verification and support.

This study’s use of a simple generic Feedback Improvement Tool (FIT) (see Additional file 3) offers a template to support the effective reflection on a suite of contextualized feedback and demonstrated the system’s utility in the training context at career outset. This study’s work has provided evidence for the introduction of the system to underpin and quality assure the support and monitoring of students who have been identified by the medical school, from whatever source, as having demonstrated lapses in their professionalism. The study also highlighted students’ appreciation and value for provided feedback and their thirst for improved systems of feedback throughout their training placements. A system based on sparse or vague feedback is open to challenge by those considered to be failing by the system. The inclusion of independent feedback in one data portfolio allowed both the quality of information provided and the response by students to be evaluated and targeted for improvement as needed. The importance of calibration of assessors highlighted by the study emphasised its importance within a successfully and reliably applied system.

### Strengths and limitations

This work further contributes to the limited evidence in this important area for both public and profession [15, 16]. Methods used in the study were robust. The focus of the study on the success in measurement of professionalism at career outset is of key importance for future research into the predictive validity of the system and its future capacity to highlight problems and remedial needs as early as possible in career.

The study had a number of limitations. Healthcare is practiced by multi-disciplinary teams and it would be useful to research the utility of *Insightful Practice* as a system to support healthcare teams in reacting to team feedback to make needed improvements. In addition, population specificity requires an ongoing programme of research in this area to build on results to date [9]. Although the literature supports *Insightful Practice* as a proxy measure for successful performance improvement, [7, 17–19] we were unable to test construct validity within this study. For example, there is evidence that well-founded and well-planned change is still a reasonable surrogate for successful implementation, [20] but it was not possible in this study to track whether students’ SMART personal objectives were carried through into future undergraduate attachments or into practice [12].

## Conclusions

The development of a system to measure and support career long professionalism in healthcare is an international challenge. This study shows the measure of *Insightful Practice* is a reliable method to identify students whose professionalism in their response to feedback is of concern and in need of remediation and monitoring. While this study uses the measurement of Insightful Practice as a surrogate for the measurement of professionalism, the reliability of the results show that students vary in their capacity to set objectives for any needed improvements even following a facilitated interview. The key point is the system offers medical schools an opportunity to both monitor students’ progress longitudinally and to help, where necessary, get students’ progress and professionalism back ‘on track’ and, where required, help inform robust decisions on suitability of students for further training. Having shown this system to be effective in established medical professionals [6] and now in medical students, the next steps will be to test the system across a multi-disciplinary group and to continue to evaluate the methods construct validity. The successful application of this study’s system, in the training context, proffers future opportunities to track student outcomes longitudinally to assess the predictive validity of the measure of *Insightful Practice* to give early warning of professional difficulties, offer early remediation and help protect patients.

## Additional file

Additional file 1: **Examples of End of Block Feedback Template**. Additional file 2:
**Example of Spot the Error Prescribing Errors Recognition Application.**
Additional file 3:
**Generic Feedback Improvement Tool (FIT).**
Additional file 4:
**Assessors Assessment of Insightful Practice Template.**
Additional file 5:
**Generalisability G- studies ANOVA tables and Components of Variance.**

## Abbreviations

AIP: Assessment of insightful practice
ANOVA: Analysis of variance
FIT: Feedback improvement tool
ICC: Intraclass correlation coefficient
Mini-CEX: Mini clinical evaluation exercise
OSCE: Objective structured clinical examinations
SMART: Specific, Measurable, Achievable, Relevant and Time based
TIPP: Tayside insightful practice portfolio
