# Supplementary figures and images for: Insightful Practice: a robust measure of medical students’ professional response to feedback on their performance
Source: BMC Med Educ. 2015 Aug 1;15:125. doi: 10.1186/s12909-015-0406-2 (PMC4522119; doi:10.1186/s12909-015-0406-2)

Additional file 1: End-of-Block Feedback Template


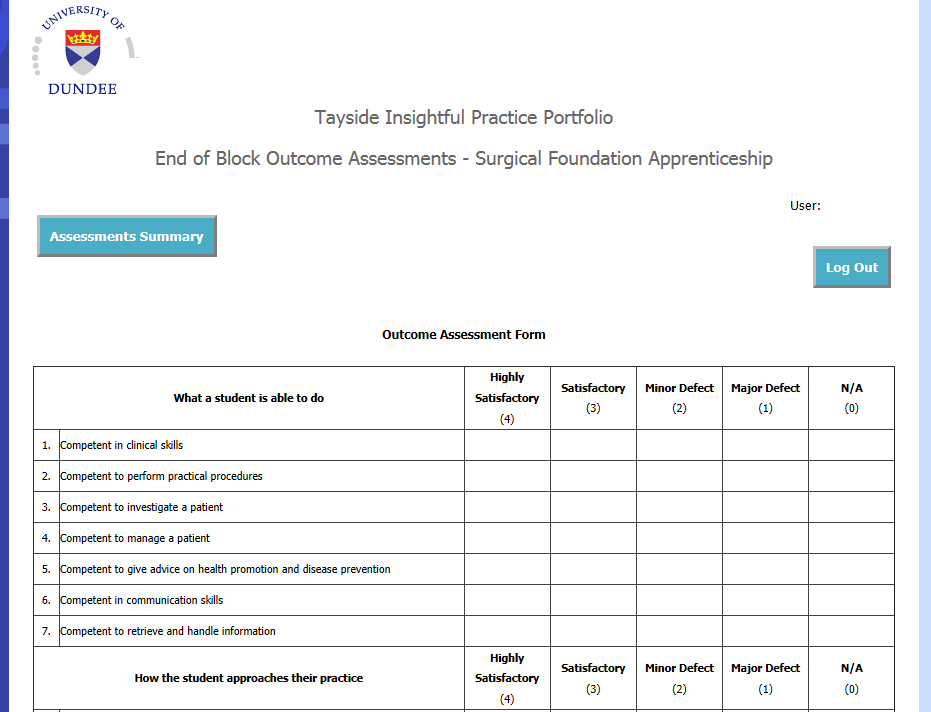

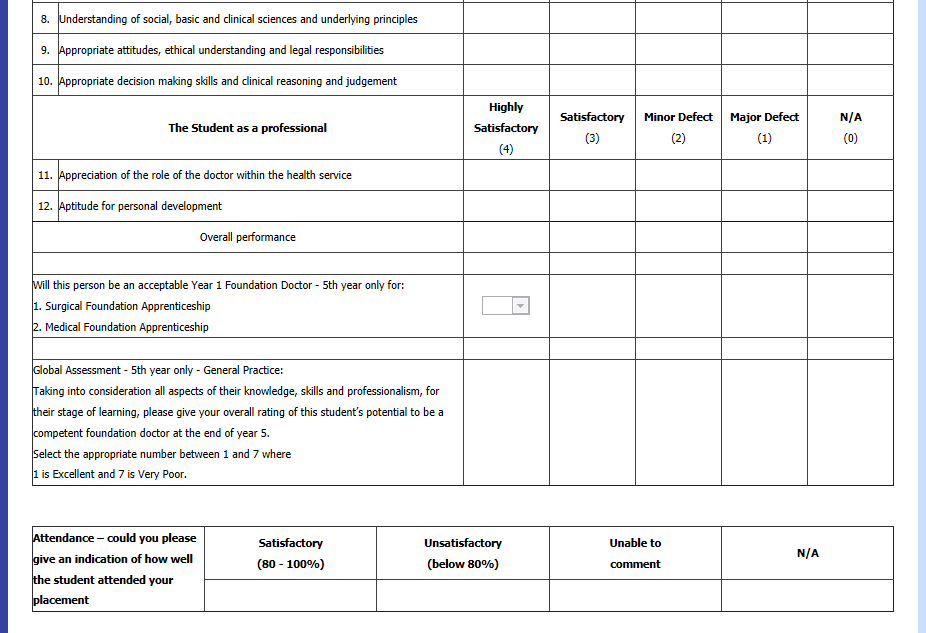


Appendix 1 cont:


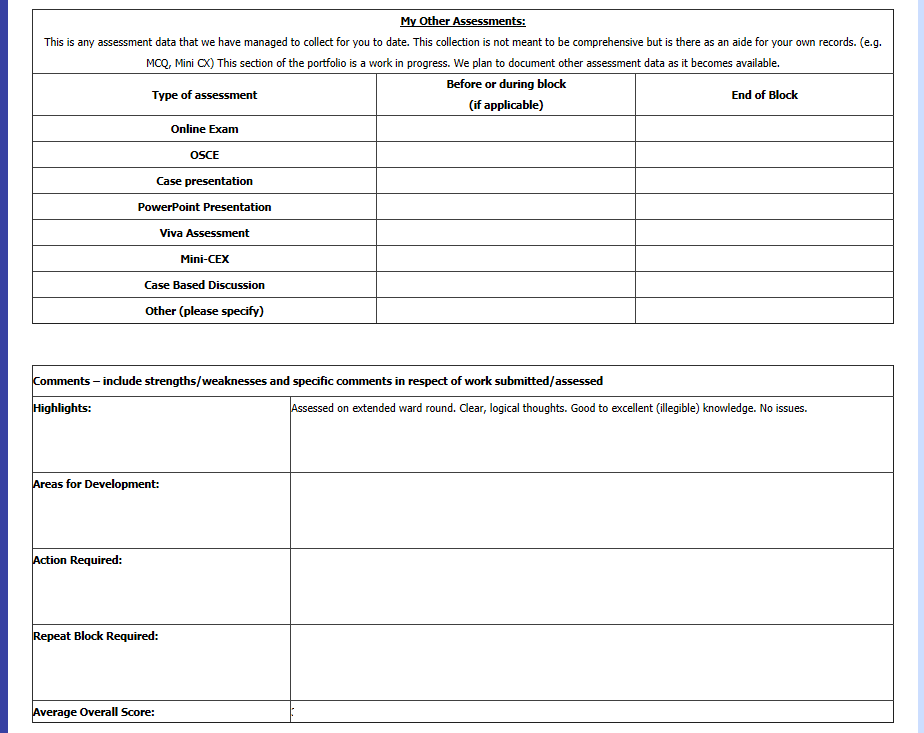


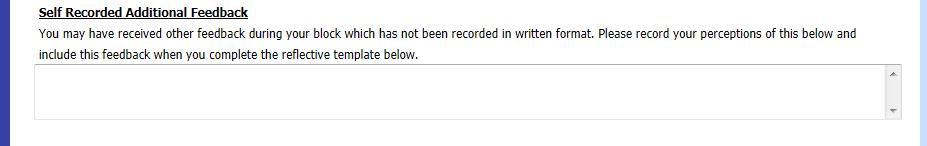

Supplement: Additional file 1: — Examples of End of Block Feedback Template. [file 12909_2015_406_MOESM1_ESM.docx]

Additional file 2: Example *Spot the Error*: Prescribing Errors Recognition Application


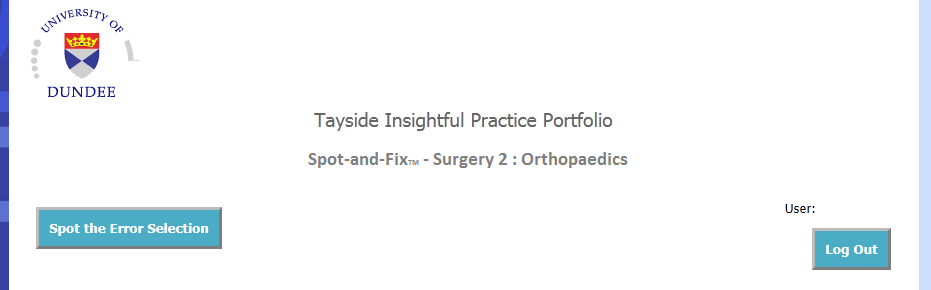

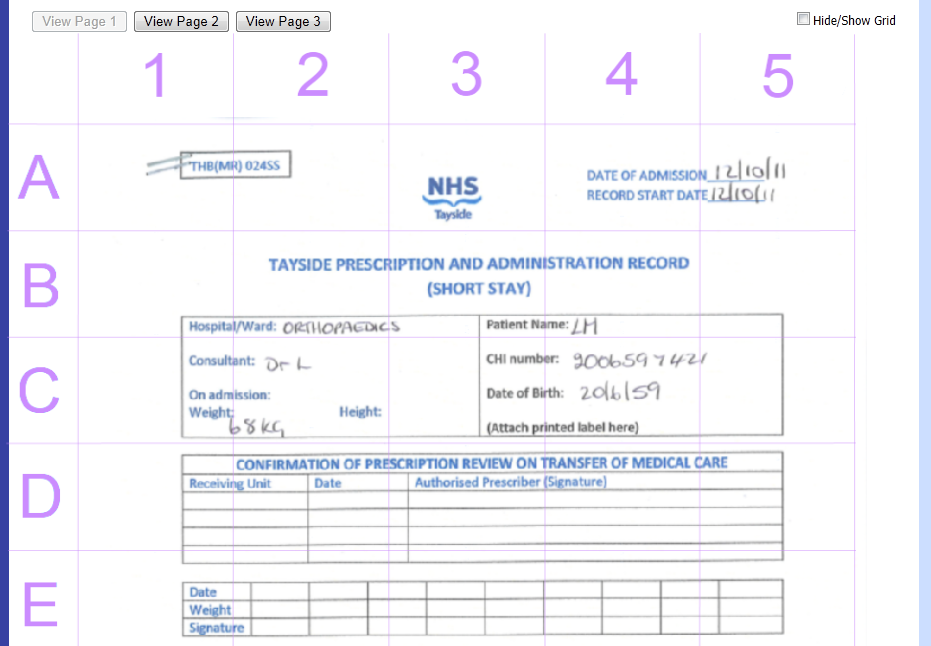


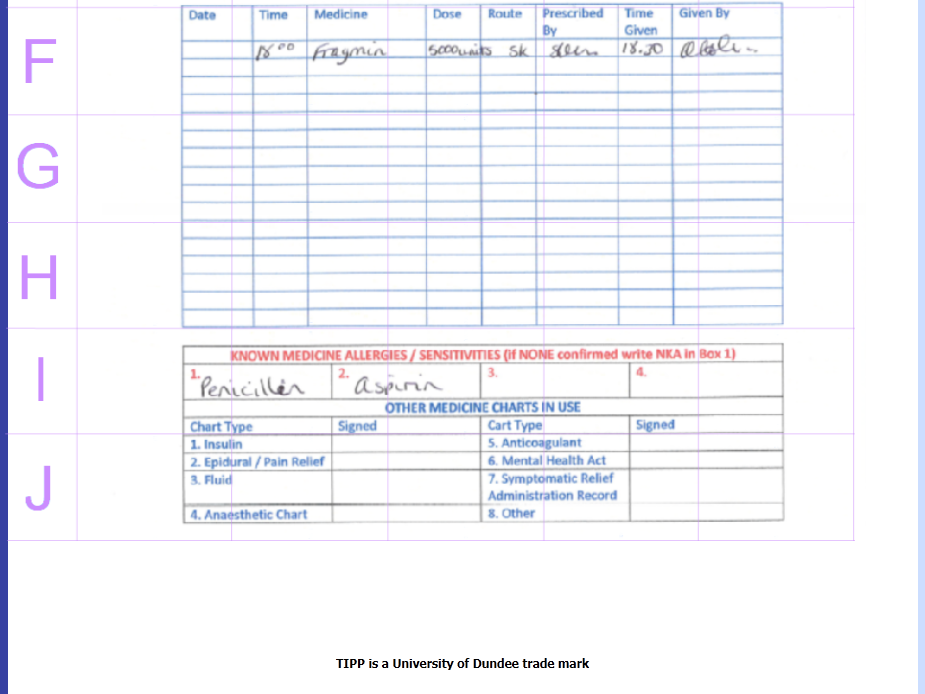


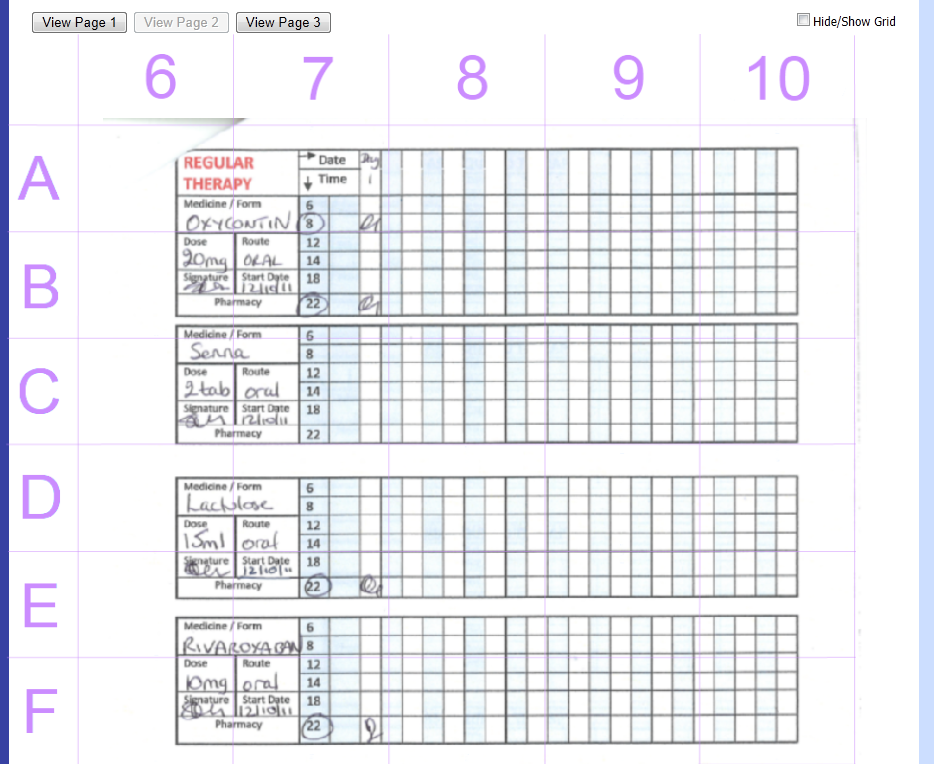

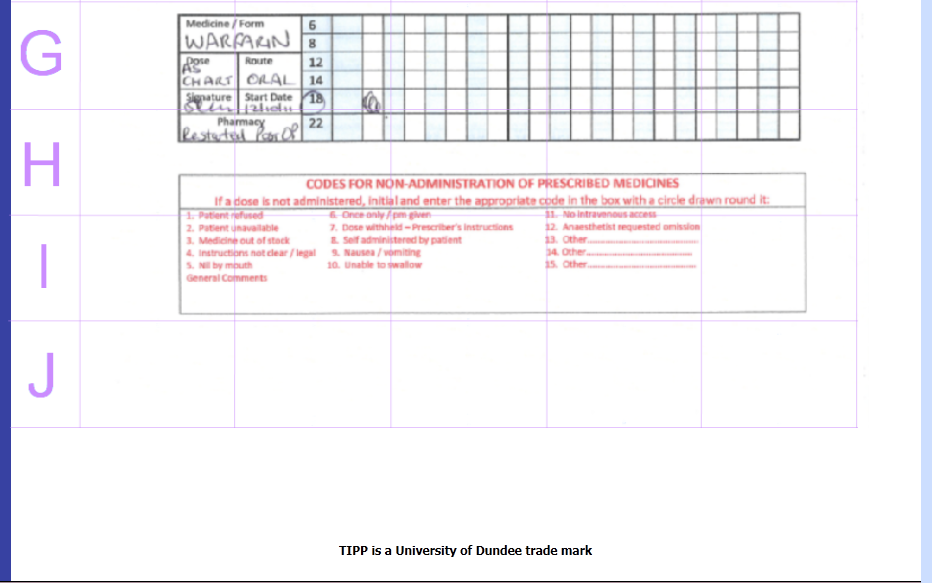


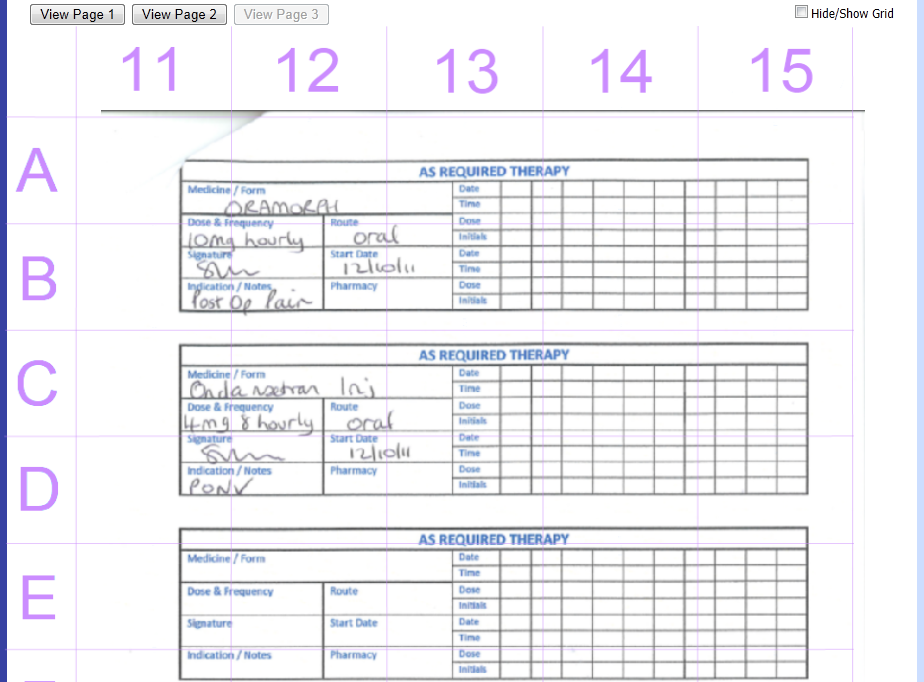

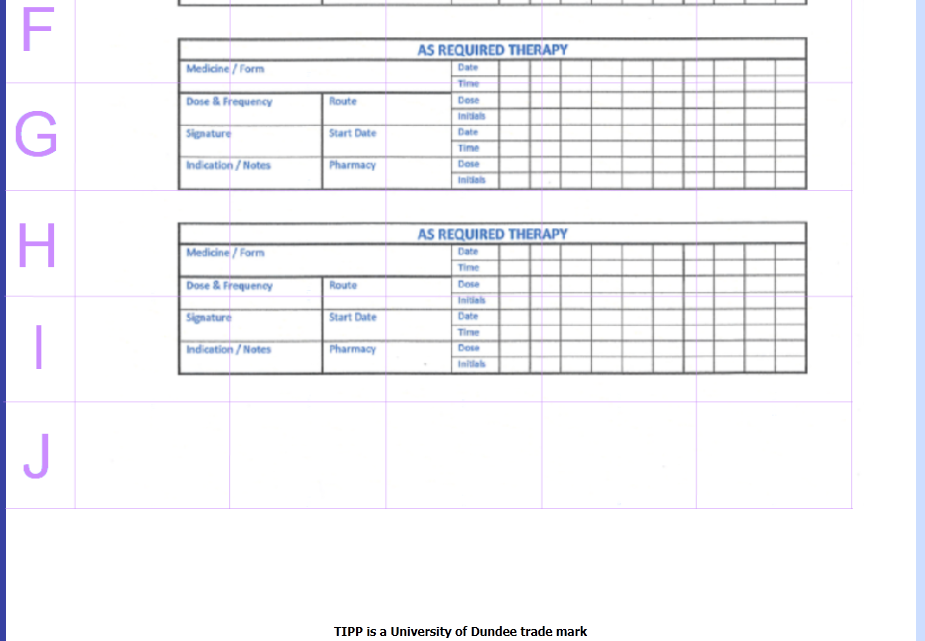


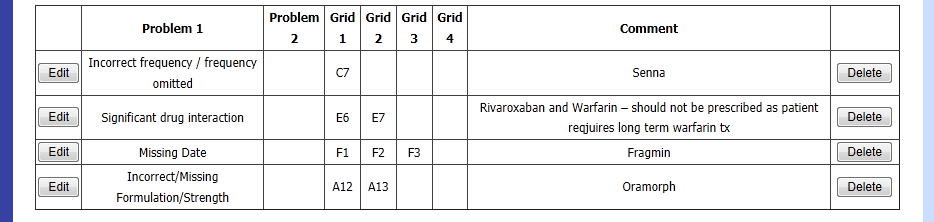

Supplement: Additional file 2: — Example of Spot the Error Prescribing Errors Recognition Application. [file 12909_2015_406_MOESM2_ESM.docx]

Additional file 3: Generic Feedback Improvement Tool (FIT)

Step1


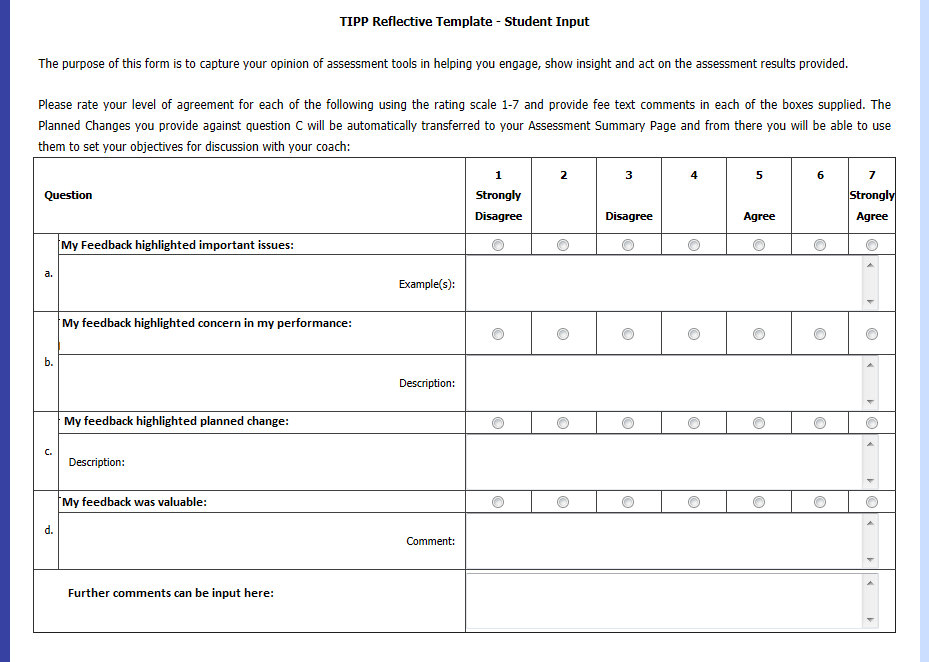


Step 2


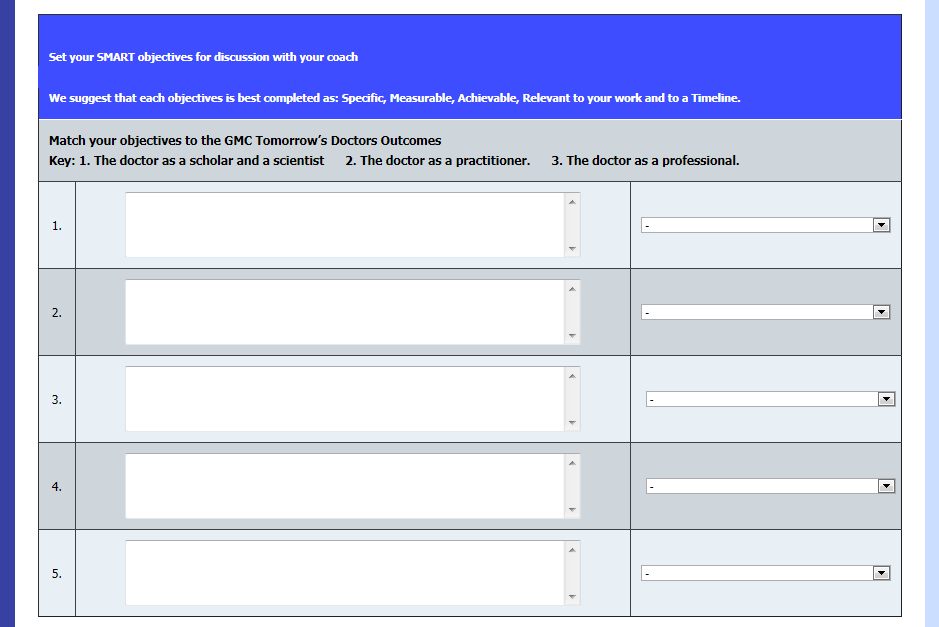


Step 3


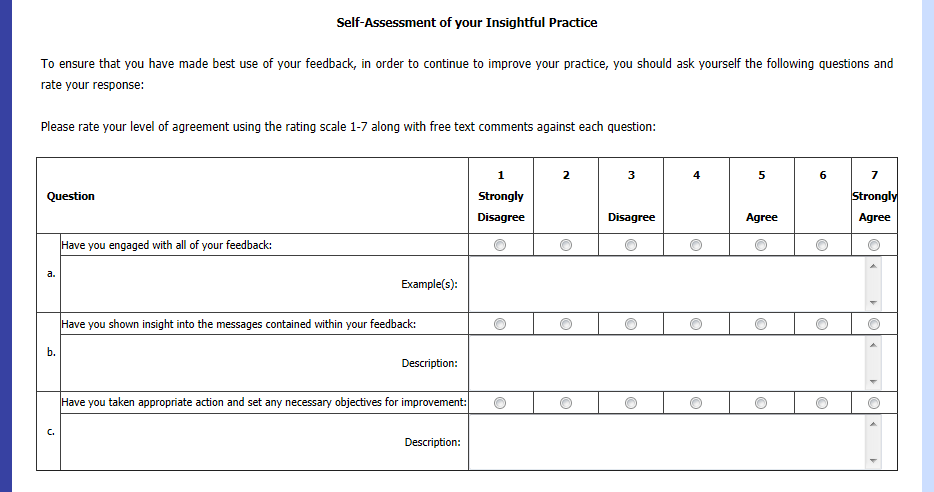

Supplement: Additional file 3: — Generic Feedback Improvement Tool (FIT). [file 12909_2015_406_MOESM3_ESM.docx]

Additional file 4: Medical School: Assessment of Insightful Practice (Students)


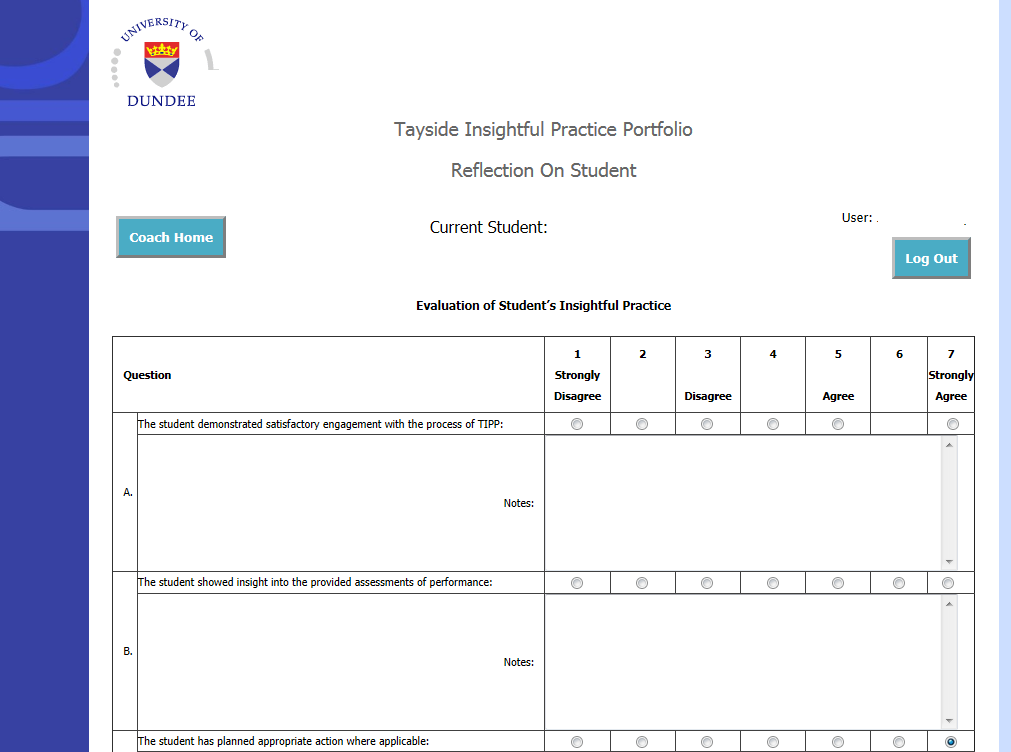


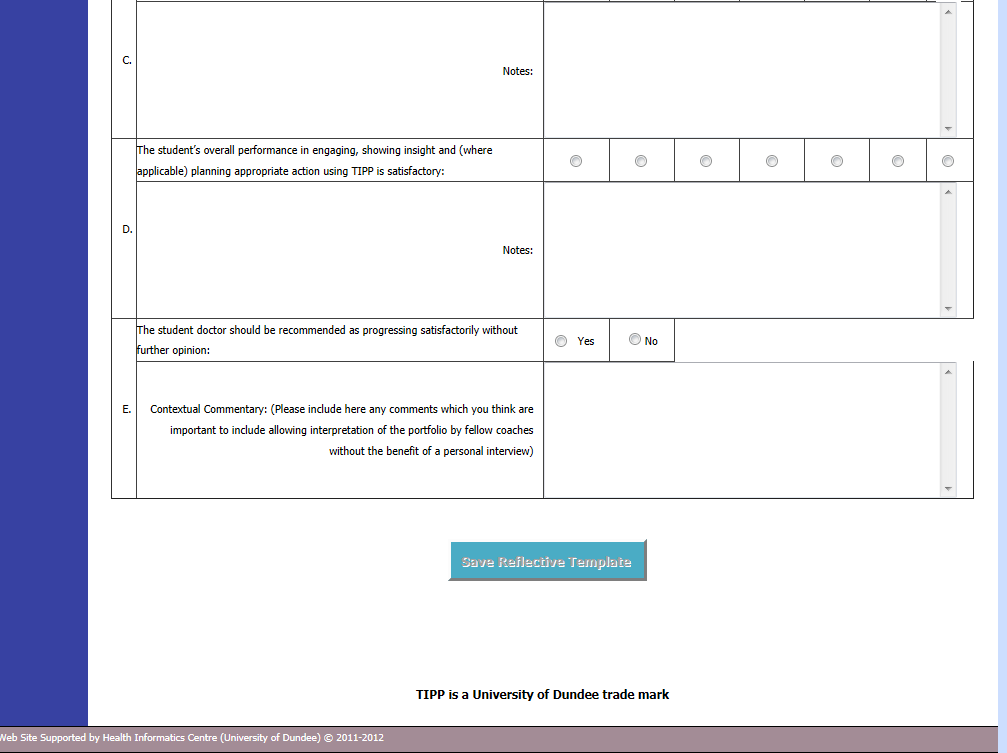

Supplement: Additional file 4: — Assessors Assessment of Insightful Practice Template. [file 12909_2015_406_MOESM4_ESM.docx]
