# Supplementary material for: Insightful Practice: a robust measure of medical students’ professional response to feedback on their performance
Source: BMC Med Educ. 2015 Aug 1;15:125. doi: 10.1186/s12909-015-0406-2 (PMC4522119; doi:10.1186/s12909-015-0406-2)
Supplement: Additional file 5: — Generalisability G- studies ANOVA tables and Components of Variance. [file 12909_2015_406_MOESM5_ESM.docx]

Additional file 5:

**Generalisability G- studies**

**ANOVA tables and Components of Variance**

Assessment of *Insightful Practice* in

4^th^ Year Medical Students

**KEY**

**Group 1**

Raters (Assessors) calibrated pre-marking (training by group marking exercise)

**Group 2**

Raters (Assessors) NOT calibrated by pre-marking (training by one-to-one individual training exercise

**Participants**

Students (S): n= 14

Rater (assessor) (R): n=3

Question (Q): n=5

**Design**

Crossed

**Analysis of variance (ANOVA) table and components of variance**

**Group 1 (Q1-3)**

| **Source** | **Degrees of Freedom** | **Sum of Squares** | **Mean Squares** | **Variance Component** |
| --- | --- | --- | --- | --- |
| **S** | 13 | 267.59523 | 20.58425 | 2.04396 |
| **R** | 2 | 15.06349 | 7.53175 | 0.14072 |
| **Q** | 2 | 2.11111 | 1.05556 | 0.00275 |
| **SR** | 26 | 40.71429 | 1.56593 | 0.43468 |
| **SQ** | 26 | 23.00000 | 0.88462 | 0.20757 |
| **RQ** | 4 | 1.26984 | 0.31746 | 0.00397 |
| **SRQ** | 52 | 13.61905 | 0.26190 | 0.26190 |

**Group 1 (Q4)**

| **Source** | **Degrees of Freedom** | **Sum of Squares** | **Mean Squares** | **Variance Component** |
| --- | --- | --- | --- | --- |
| **S** | 13 | 88.95238 | 6.84249 | 2.04212 |
| **R** | 2 | 2.71429 | 1.35714 | 0.04579 |
| **SR** | 26 | 18.61905 | 0.71612 | 0.71612 |

**Group 1 (Q5)**

| **Source** | **Degrees of Freedom** | **Sum of Squares** | **Mean Squares** | **Variance Component** |
| --- | --- | --- | --- | --- |
| **S** | 13 | 6.28571 | 0.48352 | 0.14469 |
| **R** | 2 | 0.04762 | 0.02381 | -0.00183 |
| **SR** | 26 | 1.28571 | 0.04945 | 0.04945 |

**Group 2 (Q1-3)**

| **Source** | **Degrees of Freedom** | **Sum of Squares** | **Mean Squares** | **Variance Component** |
| --- | --- | --- | --- | --- |
| **S** | 13 | 64.76984 | 4.98230 | 0.40110 |
| **R** | 2 | 29.47619 | 14.73810 | 0.31258 |
| **Q** | 2 | 2.90476 | 1.45238 | 0.02564 |
| **SR** | 26 | 38.30159 | 1.47314 | 0.37790 |
| **SQ** | 26 | 6.20635 | 0.23871 | -0.03358 |
| **RQ** | 4 | 1.90476 | 0.47619 | 0.00977 |
| **SRQ** | 52 | 17.65079 | 0.33944 | 0.33944 |

**Group 2 (Q4)**

| **Source** | **Degrees of Freedom** | **Sum of Squares** | **Mean Squares** | **Variance Component** |
| --- | --- | --- | --- | --- |
| **S** | 13 | 22.95238 | 1.76557 | 0.32784 |
| **R** | 2 | 21.00000 | 10.50000 | 0.69414 |
| **SR** | 26 | 20.33333 | 0.78205 | 0.78205 |

**Group 3 (Q5)**

| **Source** | **Degrees of Freedom** | **Sum of Squares** | **Mean Squares** | **Variance Component** |
| --- | --- | --- | --- | --- |
| **S** | 13 | 3.23810 | 0.24908 | 0.03663 |
| **R** | 2 | 1.71429 | 0.85714 | 0.05128 |
| **SR** | 26 | 3.61905 | 0.13919 | 0.13919 |

**Formulae used in calculations using components of variance (σ^2^)**

***Internal Consistency***

Internal consistency was calculated for the results based on three Raters.

G internal consistency (Questions 1-3) =

σ^2^ (Student) + σ^2^ ((Student*Rater)/3)

------------------------------------------------------------------------------------------------------------------------

σ^2^ (Student) + σ^2^ ((Student*Rater)/3) + σ^2^ ((Student*Question)/3) + σ^2^(Student*Rater*Question)/3)

***Inter-rater reliability (Questions 1-3) for number of raters (nR)***

G inter-Rater reliability =

σ^2^ (Student) + σ^2^ (Student*Question)

---------------------------------------------------------------------------------------------------------------------------

σ^2^ (Student) + σ^2^ (Student*Question) + σ^2^ ((Student*Rater)/nR) + σ^2^((Student*Rater*Question)/nR)

***Inter-Rater reliability (AIP Question 4 and Question 5) for number of Raters (nR)***

G inter-Rater reliability =

σ^2^ (Student)

--------------------------------------------

σ^2^ (Student) + σ^2^ ((Student*Rater)/nR)
